# Nasopharyngeal Development in Patients with Cleft Lip and Palate: A Retrospective Case-Control Study

**DOI:** 10.1155/2012/458507

**Published:** 2012-03-19

**Authors:** Kai Wermker, Susanne Jung, Ulrich Joos, Johannes Kleinheinz

**Affiliations:** ^1^Department of Cranio-Maxillofacial Surgery, Fachklinik Hornheide at The University of Muenster, Dorbaumstraße 300, 48157 Muenster, Germany; ^2^Research Unit Vascular Biology of Oral Structures (VABOS), Department of Cranio-Maxillofacial Surgery, University Hospital Muenster, Germany

## Abstract

*Introduction*. The aim of this paper was to evaluate cephalometrically the nasopharyngeal development of patients with complete unilateral cleft lip and palate. Influencing factors were evaluated and cleft to noncleft subjects were compared to each other. *Material and Methods*. The lateral cephalograms of 66 patients with complete cleft lip and palate were measured and compared retrospectively to the cephalograms of 123 healthy probands. Measurements were derived from a standardized analysis of 56 landmarks. *Results*. We observed significant differences between cleft and control group: the cleft patients showed amaxillary retroposition and a reduced maxillary length; the inclination of the maxilla was significantly more posterior and cranial; the anterior nasopharyngeal height was reduced; the nasopharyngeal growth followed a vertical tendency with reduced sagittal dimensions concerning hard and soft tissue. The velum length was reduced. In the cleft group, an accumulation of mandibular retrognathia and an anterior position of the hyoid were observed. Skeletal configuration and type of growth were predominantly vertical. *Conclusions*. Our data provides a fundamental radiological analysis of the nasopharyngeal development in cleft patients. It confirms the lateral cephalogram as a basic diagnostic device in the analysis of nasopharyngeal and skeletal growth in cleft patients.

## 1. Introduction

The nasopharyngeal area, with its complex interactions of bony fundament, muscular functionality, and soft tissues, influences not only the aesthetic facial harmony but also provides the anatomical basis of speech and hearing.

In patients with (unilateral) cleft lip and palate, the restoring of the velo- and nasopharyngeal function represents the crucial surgical step to a solid rehabilitation as far as phonation, articulation, and speech development are concerned.

Many morphometric studies have been performed to analyse the cephalometric characteristics of cleft patients; the causality of the altered configuration of the bony facial structures is discussed controversially.

Chatzistavrou et al. debate the influence of an inherited growth deficiency [1]. The impaired functionality of the cleaved oropharyngeal structures and their lacking potency as growth centres represents another explanation of the altered skeletal development [2–5]. Finally an iatrogenic impact on the development of the facial structures, their position, and function, after complex surgery during the first months of extra uterine development, is described [6–10].

The aim of the present investigation was to analyse the morphologic development of hard and soft tissues in the nasopharyngeal region as a pivotal anatomical region in aspects of functional rehabilitation, that is, speech, breathing, and hearing (aeration of the middle ear cavity via the tympanic tube) in a collective of cleft lip and palate patients after consistent surgical therapy in comparison to a healthy collective.

The analysis of the configuration of the skull base, characteristic growth pattern, and decisive skeletal maxillary and mandibular parameters was of special interest in this retrospective cephalometric study. This static approach does not include the correlation to functional impairment but provides an anatomical basis.

This small investigation aims to answer the following questions.

Are there significant differences concerning the configuration of the skull base between cleft and control group?
What are the most meaningful measured values in the analysis of the lateral cephalogram, differentiating cleft and control group?
What are the significant differences in the position of the velopharyngeal position?
Is there an altered position of the hyoid in the cleft group?

## 2. Material and Methods

### 2.1. Subjects

In total, we analysed the cephalometric X-rays of 66 patients with complete unilateral cleft lip, alveolus, and palate who had undergone the same operations by the same team (uCLP group). In all patients, primary closure of the lip was performed according to Millards technique [11] at the age of 6 to 8 months, and one-step closure of hard and soft palate was done according to Campbell [12] and Widmaier [13] at the age of 12 to 16 months. Age below 6 years or already performed surgery influencing the anatomy and configuration of naso-, oro-, or velopharynx, for example, velopharyngoplasty or adenoidectomy, leads to exclusion from further analysis. 

As controls served 123 healthy patients from our orthodontic department (Control group). Again patients younger than 6 years and syndromes were excluded.

### 2.2. Cephalometry

Cephalometric X-rays were made under usual standard conditions during inspiration (using cephalostats, film-focus-distance 4.0 meters, and consecutively factor of enlargement 2%, 32 mAs and depending on age and constitution 72 kV to 80 k). Digitalised X-rays showed a minimum solution of 400 dpi; analysis was performed using the software FRWIN “(Computer konkret, Systemhaus Falkenstein, Falkenstein, Germany) or for measurement of areas using Scion Image 4.0.2” (Scion Corporation, Frederick, MD, USA). 

**Figure 1:**
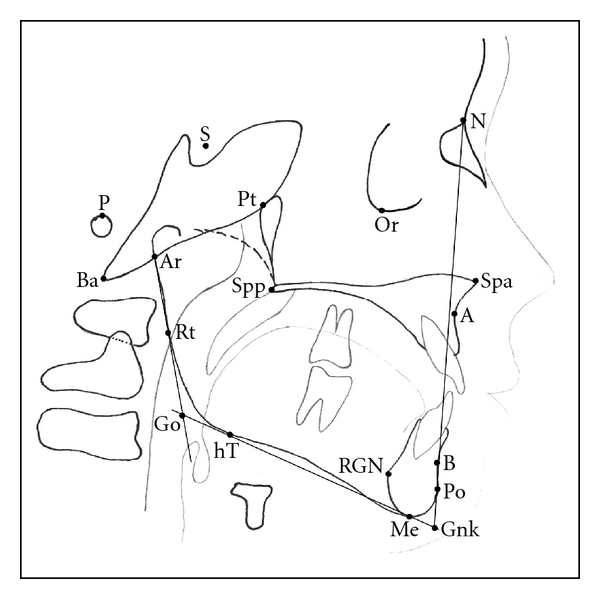
Cephalometric landmarks of the facial skeleton and skull base. (N: nasion, Or: orbitale, S: sella, P: porion, Ba: basion, Ar: articulare, Pt: pterygoid, Spp: posterior nasal spine, Spa: anterior nasal spine, A: subnasal/A-point, B: submentale/B-point, Po: pogonion, Gnk: gnathion, Me: menton, RGN: retrognathia, hT: horizontal mandibular tangential point, Rt: vertical ramus tangential point, Go: gonion).

**Figure 2:**
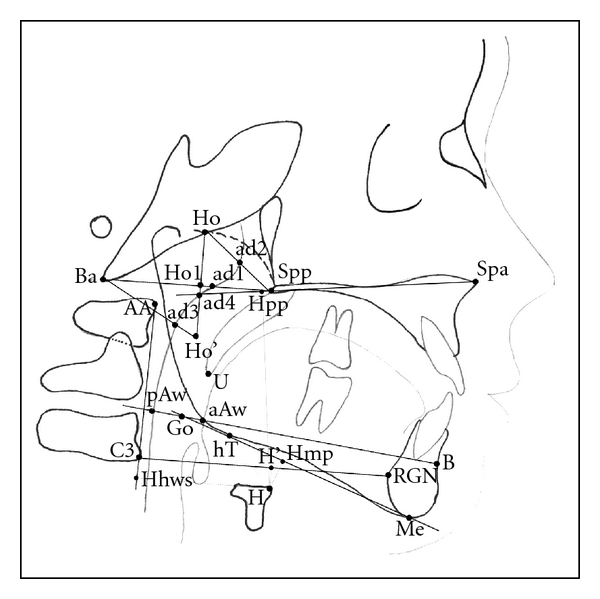
Cephalometric landmarks of the naso- and oropharyngeal area. (Spa: anterior nasal spine, Spp: posterior nasal spine, Ho: hormion, Ba: basion, AA: anterior arcus atlantis (first cervical spine), C3: anterior third cercival spine (C3), H: hyoid, Me: menton, RGN: retrognathia, B: submentale/B-point, aAw: anterior airway, pAw: posterior airway, ad1–4: adenoids 1–4).

Parameters and variables measured in this study were adopted from various previously described cephalometric analyses [14–21], with most attention to the naso-, oro-, and velopharyngeal area. Figures 1 and 2 show the relevant landmarks used in this analysis.

**Figure 3:**
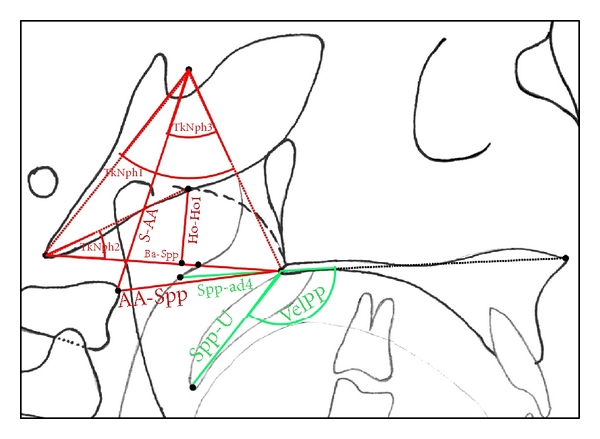
Cephalometric variables of the naso- and oropharyngeal area. (angular measurements: TkNph1: angle Ba-S-Spp, TkNph2: angle Ho-Ba-ad1, TkNph3: angle AA-S-Spp, VelPP: angle Spa-Spp-U; linear measurements are visible in this figure).

**Figure 4:**
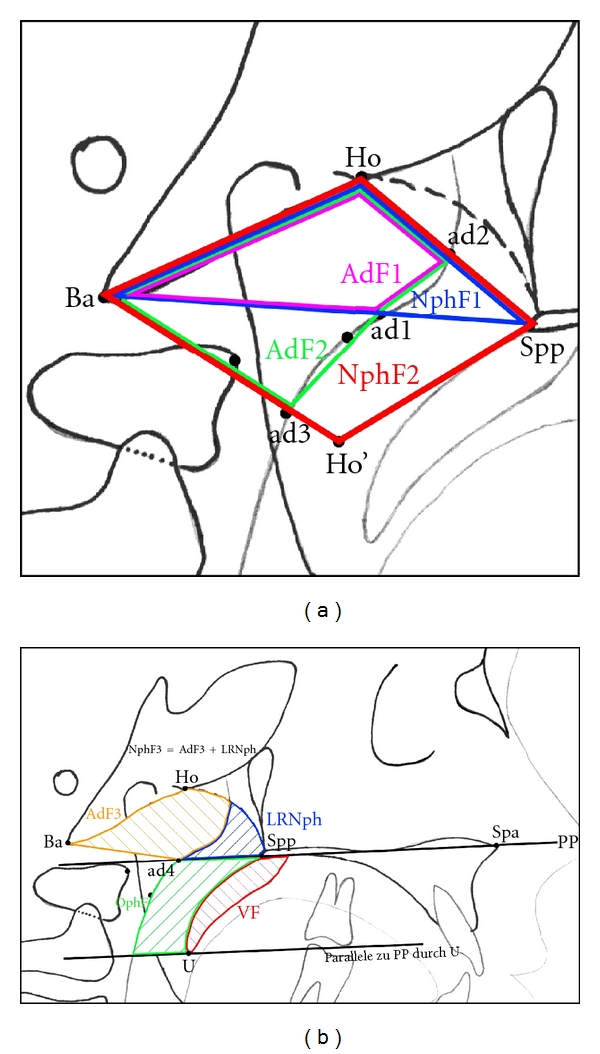
(a) (NphF1: area Spp-Ho-Ba-Spp, NphF2: area Spp-Ho-Ba-Ho′-Spp, AdF1: area ad2-Ho-Ba-ad1-ad2, AdF2: area ad2-Ho-Ba-ad3-ad1-ad2). ((a) and (b)) Measurement of areas in the naso- and oropharynx.

**Figure 5:**
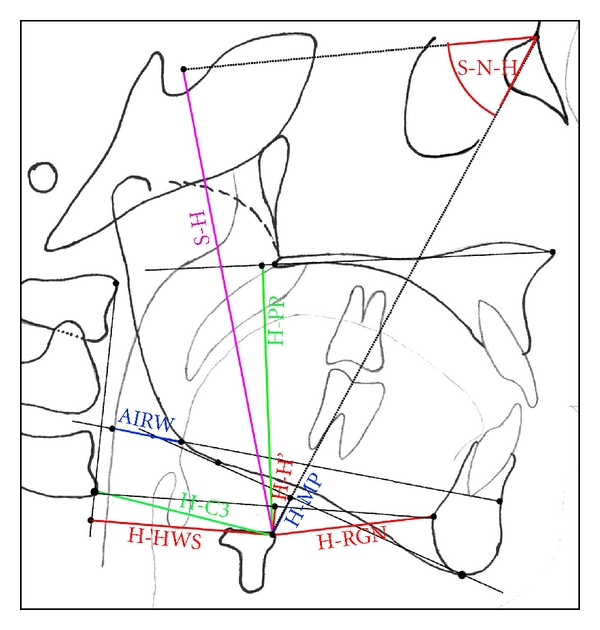
Variables measured to evaluate position of the hyoid. (angel S-N-H: hyoid position, AIRW: airway, that is, distance aAw-pAw).

**Figure 6:**
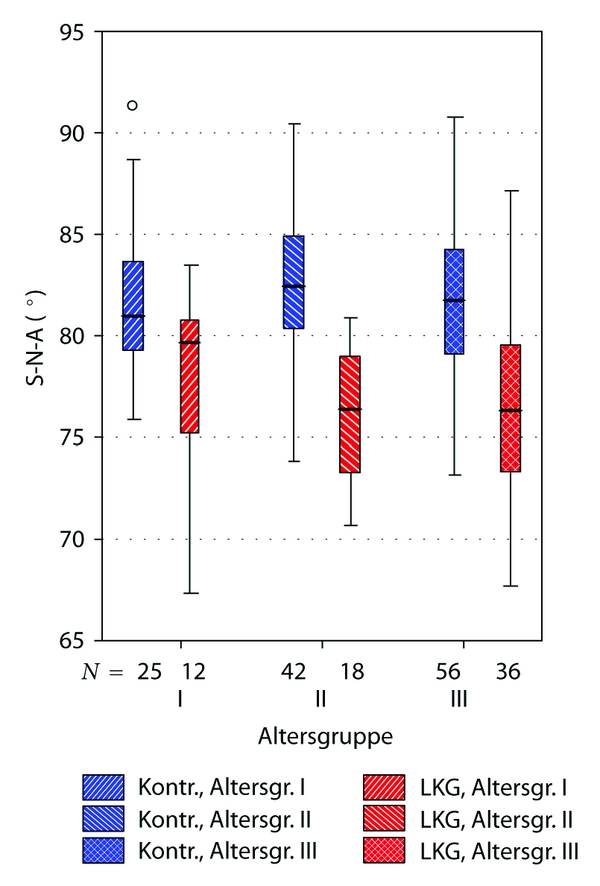
Boxplot illustrating significant differences between uCLP group and controls concerning maxillary position (angle S-N-A). “Altersgruppe”: age subgroups, explanation see Table 1. “LKG”: uCLP group (cleft). “Kontr.”: control group (noncleft).

Linear measurements were adjusted to the total length of the skull base (distance N-Ba) as internal reference to overcome confounding by different enlargement factors and to make different age groups more comparable. This technique of adjusting linear measurements to this internal reference line was also described and used by Ross [22], for example, in multicenter studies. Figures 3, 4, 5, and 6 show analysed variables of interest with regard to their anatomical region.

### 2.3. Statistical Analysis

To determine the measurement errors, 30 X-rays were randomly selected and measured twice within 2 weeks by the same examiner (KW). Randomized error according to Houston [23], combined methodology error according Dahlberg [24], and test on concordance according to the method described by Bland and Altman [25] were calculated. 

To overcome serious confounding by age, for comparison of both collectives (cleft and controls), a division into three subgroups of age as follows was done (Table 1).

Comparison of two groups (e.g., male versus female and cleft versus control) was performed using chi-square test for nominal or ordinal scaled variables; for metric variables *t*-test was used. Comparing more than two groups (e.g., comparing the three subgroups of age) was performed with ANOVA and post hoc Scheffé procedure. Correlations between variables of the maxillary region or the skull base on one side and nasopharyngeal parameters on the other side were tested on significance using Spearman's correlation analysis. 

## 3. Results

### 3.1. Error Analysis

Error analysis revealed excellent quality of measurement and good reliability. The standard deviation of 0.8 degree error in the measuring of angles and under 0.8 mm error in linear measurements documents precise analysis. Neither random errors nor systematic errors could be found. Using the Bland-Altmann procedure, except for two variables of areas (OphF and LRFV), the differences between two measurements were within the double-standard deviation, which means a good quality of the cephalometric analysis.

### 3.2. Subjects, Age, and Gender Influence

Tables 2 and 3 give an overview over age and gender distribution of included patients in both collectives. Median age was 16.4 (SD 4.1) years in the uCLP group and 15.4 (SD 6.9) years in the control group, showing no statistical significant differences between both groups concerning median age (*t*-test: *P* > 0.05) or age-subgroups (chi-square test: *P* > 0.05).

Concerning cephalometric measurements, no statistically significant differences could be found in our study population between male and female probands either in the cleft nor in the control group, although in the control group percentage of female patients was significantly higher than in the cleft group. Influence of age was certainly visible concerning linear measurements, but did not influence skeletal configuration of the face, nasopharyngeal configuration, or other relevant regions in both groups. Due to limited space, we hereby do not present the results in detail.

### 3.3. Correlations between Skull Base and Nasopharyngeal Parameters

A strong and statistical significant correlation (|*r*_*s*_| > 0.6) could be found between flexion of the skull base (angle N-S-Ba) and posterior vertical facial height (distance S-Spp) and also the anterior skull base length (distance N-S). A greater skull base flexion was associated with shorter distances S-Spp and N-S.

Clear significant correlations (|*r*_*s*_| > 0.5) could be determined between the N-S-Ba angle on the one side and angles S-N-B (position of the mandible), Ba-S-Spp (TkNph1, depth of the bony nasopharynx), and S-N-H (hyoid position).

Significant correlations with 0.4 < |*r*_*s*_| < 0.5 were observed between skull base flexion N-S-Ba and variables S-Go (total posterior facial height), GSHVER (facial height relation), and Ho-Ba-ad1 angle (TkNph2, depth of the bony nasopharynx II).

Table 4 shows the significant correlations in detail.

### 3.4. Differences between Cleft and Noncleft Probands

Table 5 shows the various differences between uCLP group and noncleft probands in detail. Patients with uCLP showed a statistically highly reduced posterior facial height (S-Spp, *P* < 0.001). Furthermore, values for angles S-N-A and Ba-N-A (position of the maxilla) and Conv-A (convexity of Point A) were highly and significantly reduced which can be interpreted as sign of maxillary retrognathia and retroposition. Also mandibular parameters differed significantly (angles S-N-B and N-Go-Me). 

In the nasopharyngeal region, vertical height (Ho-Ho1), horizontal depth (Ba-Spp), and depth of the bony nasopharynx (angle AA-S-Spp, TkNph3) were significantly diminished. Patients with uCLP showed also a reduced velar length (Spp-U) and consecutively a more unfavourable need ratio (Spp-ad4/Spp-U) (*P* < 0.01).

Comparisons between cleft and noncleft group subgrouped to three age groups (see Table 1) came to similar results. Highest significance was observed concerning the angles S-N-A (position of the maxilla), AA-S-Spp (TkNph3, depth of th bony nasopharynx), and the linear distance Ba-Spp (sagittal depth of the bony nasopharynx).

**Figure 7:**
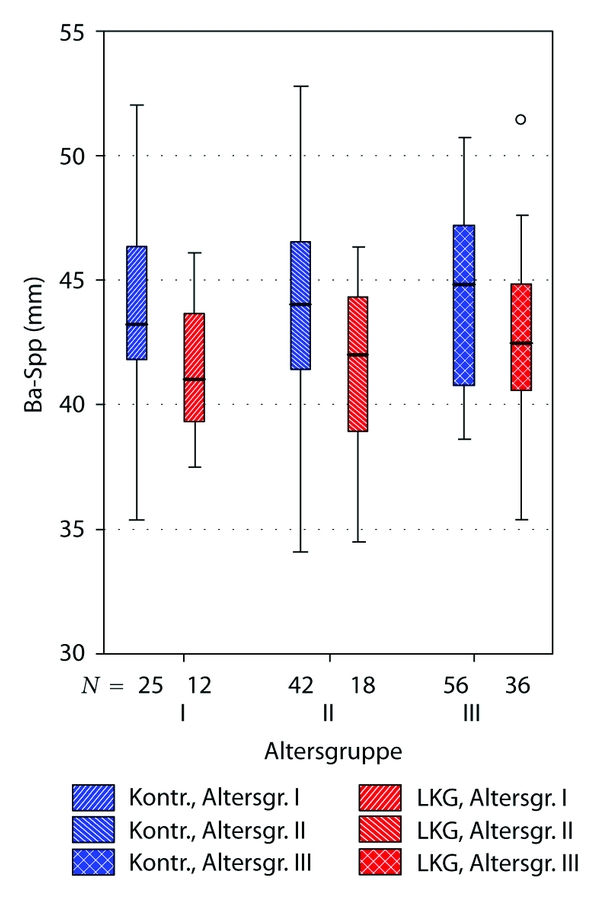
Boxplot illustrating significant differences between uCLP group and controls concerning sagittal nasopharyngeal depth (distance Ba-Spp). “Altersgruppe”: age subgroups, explanation see Table 1. “LKG”: uCLP group (cleft). “Kontr.”: control group (noncleft).

**Figure 8:**
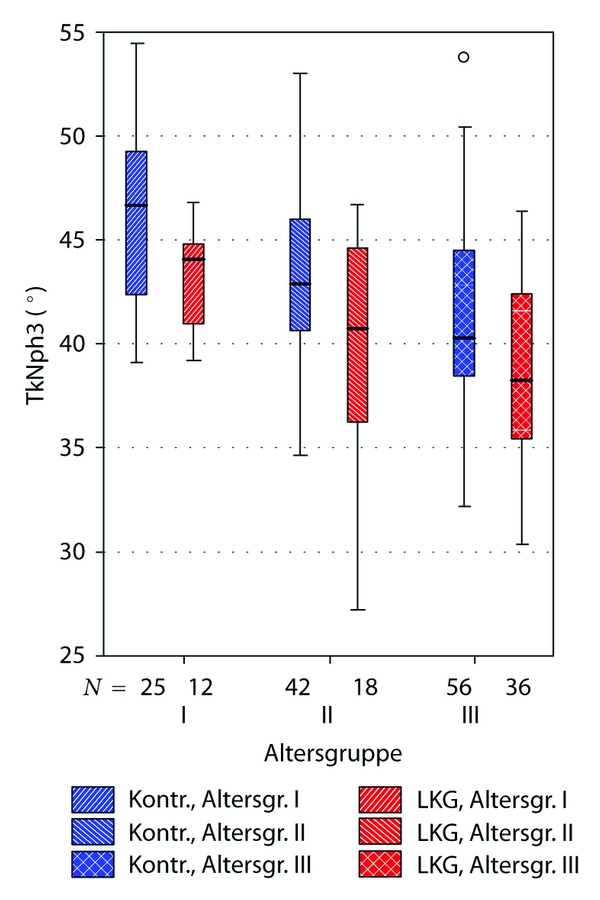
Boxplot illustrating significant differences between uCLP group and controls concerning bony nasopharyngeal depth (angle AA-S-Spp). “Altersgruppe”: age subgroups, explanation see Table 1. “LKG”: uCLP group (cleft). “Kontr.”: control group (noncleft).

The Figures 6, 7, and 8 illustrate these differences clearly.

## 4. Discussion

Generally a retrospective cephalometric analysis has to face the problem of measuring faults. There are radiographic faults, caused by the radiological technique, errors in the identification of measuring points, and errors during the appraisal of the measured points, distances, and angles.

To reduce radiological variations, all linear measurements were calibrated to the length of the scull base N-Ba according to the data of the multicentric investigations of Ross [22, 26, 27]. The standard deviation of 0.8 degree in the measuring of angles and under 0.8 mm in linear measurements stands for a high metering precision and corresponds well with the results of comparable investigations [28].

Although both compared groups showed different percentage in their male-to-female ratio, analysis revealed no statistical influence of gender on cephalometric variables as no difference could be established between males and females in this study neither in the uCLP nor in the control group. Even when keeping this missing gender matching in mind, for this reason, we assumed the control group being sufficient enough for this comparative analysis. 

We found in our data a constricted development of the midface, a reduced facial depth, a more vertical growth pattern and a reduced sagittal dimension of the nasopharyngeal complex in favour of a more vertical naso-pharyngeal development.

These results correspond with comparable findings in the literature [28–32].

The need ratio, defined as quotient of velar length and distance of the velum to the pharyngeal posterior wall, ranges in our data from 79.7% to 81.6% depending on the age of the cleft patients. The results of the control group range from 85.1% to 89.1%.

This significant difference stands for an impaired velopharyngeal closure and underlines the clinical relevance of the cephalometric measuring especially when it comes to decisions concerning surgical interventions, for example, velopharyngoplasty.

But it has to be critically kept in mind, that in our here presented cephalometric study, based on lateral X-rays of the skull, a separate assessment of velopharyngeal function and speech was not performed. Despite other studies showed clear correlations between velopharyngeal insufficiency and distinct changes concerning cephalometric measurements, this study is not able and was not designed to compare VPI and non-VPI patients [9, 31].

Which factors may have influenced the depicted differences of nasopharyngeal development and configuration in cleft patients compared to a noncleft control group? Of etiological relevance may be an impaired nasal breathing in cleft patients, postoperative scaring especially after palatal closure, and inphysiological or insufficient function of velopharyngeal muscles.

In our data, the cleft patients showed a more caudal and anterior position of the hyoid compared to a healthy collective. In addition, the hyoid position comes with greater distances to the cervical spine and the palatinal and the mandibular plane. Kaduk et al. and Rose et al. report similar results [33, 34]. In many clinical investigations, the caudal position of the hyoid is correlated to a statistical significant accumulation of nightly respiration disorders like snoring or even sleep apnoea. Additionally, these patients present frequently mandibular or maxillary retrognathia, enlarged tonsils and adenoids, and a vertical facial growth pattern. Some authors conclude that the caudal hyoid position represents a habitual adaptation to the narrower pharyngeal area [18, 35–38].

The lateral cephalometry as a two-dimensional analysis provides enough meaningful information for an effectual appraisal not only of the skeletal skull but also of the nasopharyngeal area [39]. It does not include the transversal dimension and neglects, therefore influencing factors like a deviated nasal septum or functional aspects like mobility of the velum and velopharyngeal closure mechanism, but it represents a low price and convenient routine diagnostic device in the analysis of skeletal and soft tissue landmarks.

## 5. Conclusions

The cephalometric comparison of cleft patients to a healthy control group showed significant differences: due to an impaired ventrocaudal growth tendency of the naso-maxillary area a retroposition of the maxillary complex in combination with a reduced maxillary length and height, especially in the area of the posterior nasal spine, was detected. In the cleft group, a reduced sagittal dimension of the hard and soft tissues of the nasopharyngeal complex in favour of a more accentuated vertical development became obvious. Combined with a reduced velar length, the result is an insufficient velopharyngeal closure.

In the cleft collective, a cumulation of mandibular retrognathia and a more caudal and anterior position of the hyoid was observed.

The results underline the pivotal role of the functional reconstruction of the velo-pharyngeal muscles to allow not only for a physiological speech development but also for a regular growth of the naso-, oro-, and velopharyngeal structures.

## Figures and Tables

**Table 1:** Definition of subgroups based on age.

Age at taking cephalometric X-ray	Age Subgroup
6–11 years	I
>11–16 years	II
>16 years	III

**Table 2:** Cleft group (explanation of age-subgroups see Table 1).

Age subgroup	Gender				Total	
	Female		Male	
	*n*	%	*n*	%	*n*	%
I	4	6.1%	8	12.1%	12	18.2%
II	8	12.1%	10	15.2%	18	27.3%
III	11	16.7%	25	37.9%	36	54.5%

Total	23	34.8%	43	65.2%	66	100.0%

**Table 3:** Control group (for explanation of age-subgroups, see Table 1).

Age subgroup	Gender				Total	
	Female		Male	
	*n*	%	*n*	%	*n*	%
I	11	8.9%	14	11.4%	25	20.3%
II	20	16.3%	22	17.9%	42	34.1%
III	44	35.8%	12	9.8%	56	45.5%

Total	75	61.0%	48	39.0%	123	100.0%

**Table 4:** Significant correlations found between skull base flexion and other cephalometric variables.

Correlation of N-S-Ba to	N-S	N-S	S-Go	GSHVER
Correlation coefficient *r*_*s*_	−0.645**	−0.645**	−0.401**	0.414**

Correlation of N-S-Ba to	S-N-A	Conv-A	S-N-B	S-AA
Correlation coefficient *r*_*s*_	−0.344**	0.294**	−0.537**	−0.346**

Correlation of N-S-Ba to	Ho-Ho1	TkNph1	TkNph2	TkNph3
Correlation coefficient *r*_*s*_	−0.322**	0.518**	−0.472**	0.336**

Correlation of N-S-Ba to	H-S	S-N-H		
Correlation coefficient *r*_*s*_	−0.342**	−0.535**		

*r*_*s*_: Spearman's correlation coefficient.

Level of significance: *P < 0.05, **P < 0.01).

(GSHVER = facial height relation = S-Go/N-Me, Conv-A = convexity of point A = distance of A-point perpendicular to N-Po; other variables see Figures 1–5).

**Table 5:** Significant differences between uCLP and control group in total.

Variable	Unit		Group		
			uCLP	uCLP	Sign.
			*n* = 66	*n* = 123	
FACAX	°	mean	88.1	90.7	**
		SD	6.8	4.7	

N-BA	mm	mean	101.2	97.9	**
		SD	6.2	6.7	

N-S	mm	mean	67.2	68.3	*
		SD	2.3	2.6	

S-Spp	mm	mean	43.9	46.4	***
		SD	3.1	4.8	

A-Me	mm	mean	59.7	58.1	*
		SD	4.7	4.9	

N-Me	mm	mean	113.8	111.6	*
		SD	7.0	7.1	

GSHVER	%	mean	64.0	65.8	*
		SD	5.6	5.3	

Spp-A	mm	mean	43.5	44.9	**
		SD	3.5	2.8	

Spp-Spa	mm	mean	47.2	48.3	*
		SD	3.3	2.9	

S-N-A	°	mean	76.7	82.0	***
		SD	4.5	3.9	

Ba-N-A	°	mean	58.2	63.4	***
		SD	4.8	3.6	

Conv-A	mm	mean	−0.6	2.2	***
		SD	4.0	2.9	

S-N-B	°	mean	76.0	78.5	***
		SD	4.3	4.7	

Ar-Go-Me	°	mean	129.1	125.9	**
		SD	6.9	7.8	

N-Go-Me	°	mean	76.5	72.5	***
		SD	6.8	5.3	

FACDEP	°	mean	83.6	85.6	*
		SD	5.6	4.0	

MANPLA	°	mean	30.1	26.3	**
		SD	9.0	6.0	

S-AA	mm	mean	51.3	49.1	**
		SD	5.9	4.5	

Ho-Ho1	mm	mean	17.5	16.2	***
		SD	2.2	1.5	

Ba-Spp	mm	mean	41.3	44.8	***
		SD	4.1	3.6	

AA-Spp	mm	mean	33.4	35.3	**
		SD	4.5	3.2	

TkNph1	°	mean	58.1	61.1	**
		SD	5.8	4.6	
TkNph3	°	mean	39.8	43.1	***
		SD	5.0	4.6	

NphF1	mm^2^	mean	375.4	349.7	*
		SD	69.9	56.7	

NphF2	mm^2^	mean	750.8	698.9	*
		SD	139.7	119.0	

AdF1	mm^2^	mean	231.2	212.4	*
		SD	44.4	45.0	

AdF2	mm^2^	mean	363.2	335.8	*
		SD	76.6	76.7	

Spp-U	mm	mean	29.1	31.8	***
		SD	5.0	3.4	

Spp-ad4	mm	mean	22.9	27.5	***
		SD	3.7	3.3	

NeedRat	%	mean	80.8	87.3	*
		SD	18.8	13.2	

H-C3	mm	mean	34.4	32.4	**
		SD	4.7	4.5	

H-MP	mm	mean	19.0	17.0	*
		SD	6.1	5.6	

H-PP	mm	mean	60.5	58.0	*
		SD	7.3	6.5	

H-HWS	mm	mean	32.9	30.8	**
		SD	4.4	4.2	

Level of significance (*t*-test): *P < 0.05, **P < 0.01, ***P < 0.001 (FACAX = facial axis = angle between Ba-N and Pt-Gnk, GSHVER = facial height relation = S-Go/N-Me, CONV-A = convexity of point A = distance of A-point perpendicular to N-Po, FACDEP = facial depth = angle between P-Or and N-Po, MANPLA = mandibular plane = angle between P-Or and hT-Me, NEEDRAT = need ratio = Spp-ad4/Spp-U, other variables see Figures 1–5).

## References

[1] Chatzistavrou E, Ross RB, Tompson BD, Johnston MC (2004). Predisposing factors to formation of cleft lip and palate: inherited craniofacial skeletal morphology. *Cleft Palate-Craniofacial Journal*.

[2] Joos U (1987). The importance of muscular reconstruction in the treatment of cleft lip and palate. *Scandinavian Journal of Plastic and Reconstructive Surgery*.

[3] Joos U (1995). Die Behandlung kranio-fazialer Anomalien. *Dtsch Z Mund Kiefer Gesichtschir*.

[4] Joos U (1995). Skeletal growth after muscular reconstruction for cleft lip, alveolus, and palate. *British Journal of Oral and Maxillofacial Surgery*.

[5] Löhle E, Joos U, Göz G, Pfeifer G (1991). Phoniatrics results following reconstruction of Palatoglossus and Palatopharyngeus muscles. *Craniofacial Abnormalities and Clefts of the Lip, Alveolus and Palate*.

[6] Rose E, Thissen U, Otten JE, Jonas I (2003). Cephalometric assessment of the posterior airway space in patients with cleft palate after palatoplasty. *Cleft Palate-Craniofacial Journal*.

[7] Crabb JJ, Foster TD (1977). Growth defects in unrepaired unilateral cleft lip and palate. *Oral Surgery Oral Medicine and Oral Pathology*.

[8] Mazahery M, Krogman WM, Harding RL (1977). Longitudinal analysis of growth of the soft palate and nasopharynx from six months to six years. *Cleft Palate Journal*.

[9] Satoh K, Wada T, Tachimura T, Fukuda J (2005). Velar ascent and morphological factors affecting velopharyngeal function in patients with cleft palate and noncleft controls: a cephalometric study. *International Journal of Oral and Maxillofacial Surgery*.

[10] Smahel Z, Kasalova P, Skvarilova B (1991). Morphometric nasopharyngeal characteristics in facial clefts. *Journal of Craniofacial Genetics and Developmental Biology*.

[11] Millard DR (1958). Columella lengthening by a forked flap. *Plastic and reconstructive surgery*.

[12] Campbell A (1926). The closure of congenital clefts of the hard palate. *British Journal of Surgery*.

[13] Widmaier W (1959). A new technic for closure of cleft palate. *Der Chirurg; Zeitschrift für alle Gebiete der operativen Medizen*.

[14] Ehmer U, Wegener H, Mende C, Dörr-Neudeck K

[15] Rakosi T (1988). *Atlas und Anleitung zur Praktischen Fernröntgenanalyse*.

[16] Linder-Aronson S (1970). Adenoids: their effect on mode of breathing and nasal airflow and their relationship to characteristics of the facial skeleton and the denition. A biometric, rhino-manometric and cephalometro-radiographic study on children with and without adenoids. *Acta Oto-Laryngologica*.

[17] Linder Aronson S, Henrikson CO (1973). Radiocephalometric analysis of anteroposterior nasopharyngeal dimensions in 6 to 12 yr old mouth breathers compared with nose breathers. *Journal for Oto-Rhino-Laryngology*.

[18] Lowe AA, Ono T, Ferguson KA, Pae EK, Ryan CF, Fleetham JA (1996). Cephalometric comparisons of craniofacial and upper airway structure by skeletal subtype and gender in patients with obstructive sleep apnea. *American Journal of Orthodontics and Dentofacial Orthopedics*.

[19] Figueroa AA, Glupker TJ, Fitz MG, BeGole EA (1991). Mandible, tongue, and airway in Pierre Robin sequence: a longitudinal cephalometric study. *Cleft Palate-Craniofacial Journal*.

[20] Rocabado M (1983). Biomechanical relationship of the cranial, cervical, and hyoid regions. *The Journal of Cranio-Mandibular Practice*.

[21] Fiedler F (1990). *Die Entwicklung im nasopharyngealen Bereich bei Patienten mit einseitigen Lippen-, Kiefer- und Gaumenspalten—eine röntgenkephalometrische Langzeitstudie*.

[22] Ross RB (1987). Treatment variables affecting growth in cleft lip and palate: part 1–7. *Cleft Palate Journal*.

[23] Houston WJB (1983). The analysis of errors in orthodontic measurements. *American Journal of Orthodontics*.

[24] Dahlberg G (1940). *Statistical Methods for Medical and Biological Students*.

[25] Bland JM, Altman DG (1986). Statistical methods for assessing agreement between two methods of clinical measurement. *The Lancet*.

[26] Ross RB Growth prediction in cleft lip and palate.

[27] Ross RB (1995). Growth of the facial skeleton following the Malek repair for unilateral cleft lip and palate. *Cleft Palate-Craniofacial Journal*.

[28] Stellzig-Eisenhauer A (2001). The influence of cephalometric parameters on resonance of speech in cleft lip and palate patients: an interdisciplinary study. *Journal of Orofacial Orthopedics*.

[29] Jonas I, Joos U, Mann W, Fiedler F, Schilli W, Rakosi T, Pfeifer G (1990). Nasopharyngeal growth following surgical treatment in unilateral cleft lip and palate cases: a long-term study on lateral cephalometric radiographs. *Craniofacial Abnormalities and Clefts of Lip, Alveolus and Palate*.

[30] Satoh K, Wada T, Tachimura T, Sakoda S, Shiba R (1998). A cephalometric study by multivariate analysis of growth of the bony nasopharynx in patients with clefts and non-cleft controls. *Journal of Cranio-Maxillo-Facial Surgery*.

[31] Satoh K, Wada T, Tachimura T, Shiba R (2002). The effect of growth of nasopharyngeal structures in velopharyngeal closure in patients with repaired cleft palate and controls without clefts: a cephalometric study. *British Journal of Oral and Maxillofacial Surgery*.

[32] Smahel Z, Mullerova I (1992). Nasopharyngeal characteristics in children with cleft lip and palate. *Cleft Palate-Craniofacial Journal*.

[33] Kaduk WMH, Grabowski R, Gundlach KKH (2003). Position of the hyoid bone in cleft lip, alveolus, and palate: variation of normal anatomy or sign accompanying the malformation?. *Cleft Palate-Craniofacial Journal*.

[34] Rose E, Thissen U, Otten JE, Jonas I (2003). Cephalometric assessment of the posterior airway space in patients with cleft palate after palatoplasty. *Cleft Palate-Craniofacial Journal*.

[35] Behlfelt K, Linder-Aronson S, McWilliam J, Neander P, Laage-Hellman J (1990). Cranio-facial morphology in children with and without enlarged tonsils. *European Journal of Orthodontics*.

[36] Behlfelt K, Linder-Aronson S, Neander P (1990). Posture of the head, the hyoid bone, and the tongue in children with and without enlarged tonsils. *European Journal of Orthodontics*.

[37] Lowe AA, Özbek MM, Miyamoto K, Pae E-K (1997). Cephalometric and demographic characteristics of obstructive sleep apnea: an evaluation with partial least squares analysis. *Angle Orthodontist*.

[38] Ozbek MM, Miyamoto K, Lowe AA, Fleetham JA (1998). Natural head posture, upper airway morphology and obstructive sleep apnoea severity in adults. *European Journal of Orthodontics*.

[39] Holmberg H, Linder-Aronson S (1979). Cephalometric radiographs as a means of evaluating the capacity of the nasal and nasopharyngeal airway. *American Journal of Orthodontics*.

